# Enhancing glioblastoma cytotoxicity through encapsulating O6-benzylguanine and temozolomide in PEGylated liposomal nanocarrier: an in vitro study

**DOI:** 10.1007/s13205-024-04123-2

**Published:** 2024-10-23

**Authors:** Manasa Manjunath Hegde, Pranoti Palkar, Sadhana P. Mutalik, Srinivas Mutalik, Jayant Sastri Goda, B. S. Satish Rao

**Affiliations:** 1 Department of Radiation Biology & Toxicology, Manipal School of Life Sciences, Manipal Academy of Higher Education, Manipal, India; 2 Advance Centre for Treatment Research and Education in Cancer, Tata Memorial Centre & Homi Bhaba National Institute, Navi Mumbai, India; 3 Manipal College of Pharmaceutical Sciences, Manipal Academy of Higher Education, Manipal, India; 4 Manipal School of Life Sciences & Director-Research, Manipal Academy of Higher Education, Manipal, India; 5 Department of Radiation Oncology, Advanced Centre for Treatment Research Education in Cancer, Tata Memorial Centre & Homi Bhaba National Institute, Navi Mumbai, India

**Keywords:** Glioblastoma multiforme, Liposomes, Temozolomide, O6-benzylguanine, Combination therapy

## Abstract

Glioblastoma (GBM) (grade IV glioma) is the most fatal brain tumor, with a median survival of just 14 months despite current treatments. Temozolomide (TMZ), an alkylating agent used with radiation, faces challenges such as systemic toxicity, poor absorption, and drug resistance. To enhance TMZ effectiveness, we developed poly(ethylene glycol) (PEG) liposomes co-loaded with TMZ and O6-benzylguanine (O6-BG) for targeted glioma therapy. These liposomes, prepared using the thin-layer hydration method, had an average size of 146.33 ± 6.75 nm and a negative zeta potential (−49.6 ± 3.1 mV). Drug release was slower at physiological pH, with 66.84 ± 4.62% of TMZ and 69.70 ± 2.88% of O6-BG released, indicating stability at physiological conditions. The liposomes showed significantly higher cellular uptake (p < 0.05) than the free dye. The dual drug-loaded liposomes exhibited superior cytotoxicity against U87 glioma cells, with a lower IC_50_ value (3.99µg/mL) than the free drug combination, demonstrating enhanced anticancer efficacy. The liposome formulation induced higher apoptosis (19.42 ± 3.5%) by causing sub-G0/G1 cell cycle arrest. The novelty of our study lies in co-encapsulating TMZ and O6-BG within PEGylated liposomes, effectively overcoming drug resistance and improving targeted delivery for glioma treatment.

**Supplementary Information:**

The online version contains supplementary material available at 10.1007/s13205-024-04123-2.

## Introduction

Glioblastomas are malignant tumors that account for 70% of all adult brain tumors and have an aggressive and morbid natural history with a high propensity for disease relapse or progression. The World Health Organization classified glioma as a grade IV tumor with a global incidence of approximately 10 per 100,000 people. Glioma patients have a poor prognosis, with a median survival of approximately 14–18 months, even with the best treatment modalities available in the contemporary era, making these malignant tumors among the most difficult to treat and thereby posing critical public health issues (Grochans et al. 2022). The current standard treatment modality for glioblastoma is maximal and safe resection via surgery followed by radiotherapy and chemotherapy. TMZ, an alkylating agent that is lipophilic in nature (Delello Di Filippo et al. 2021; Newlands et al. 1997; Zhang et al. 2012), is the present gold-standard chemotherapeutic agent that has been shown to have an anti-glioma effect (Newlands et al. 1992). The majority of GBM cells are resistant to TMZ therapy regardless of their ability to cross the blood–brain barrier (BBB) due to the activation of direct repair mechanisms via methyl guanine methyl transferase (MGMT), low mismatch repair mechanisms, changes in the cell cycle, and ATP-dependent drug efflux mechanisms (Fan et al. 2013). Furthermore, TMZ use is linked to unwanted toxicity in normal tissues (Friedman et al. 2000a). Targeting the mechanisms of chemotherapy resistance is a novel approach for combating this lethal disease. Since MGMT has been linked to tumor cell resistance to TMZ, it is possible that targeting this DNA repair protein can help restore chemotherapy activity (Hegi et al. 2004; Yu et al. 2020). O6-BGis one of the most extensively studied irreversible inhibitors of the enzyme MGMT and is known to enhance the cytotoxic effect of alkylating chemotherapeutics such as TMZ (Wedge and Newlands 1996). O6-BG is a small molecule that can easily pass through the BBB. O6-BG acts as a pseudosubstrate by adding a benzyl group to the cysteine 145 residue of the MGMT active site, thereby inactivating MGMT and preventing the removal of methyl groups from DNA (Bobola et al. 2005; Koch et al. 2007; Pegg 2000). Many studies have been conducted on the use of O6-BG with presently authorized therapeutic interventions. In a phase I trial of recurrent malignant glioma, the combination of O6-BG with carmustine wafers resulted in the infusion of O6-BG at a dose of 120 mg/m^2^ for 1 h, followed by a continuous infusion of carmustine at 30 mg/m^2^/day for 6 weeks, which reduced the dose-limiting toxicity of the drugs. In phase II trials, no benefit of using O6-BG was observed (Friedman et al. 2000b; Quinn et al. 2002). The ability of O6-BG to restore TMZ sensitivity in TMZ-resistant glioblastoma has also been studied. In a phase II clinical trial, O6-BG was combined with TMZ to assess its role in restoring TMZ sensitivity with reduced systemic toxicity. However, only 16% of patients with resistant high-grade gliomas respond to therapy. The combination of O6-BG with TMZ showed limited efficacy in this trial, as evidenced by the low response rate. While the strategy of using O6-BG to inhibit MGMT to increase TMZ sensitivity is promising, the clinical results suggest that this combination may not be sufficient to achieve significant therapeutic benefits. Therefore, tackling TMZ resistance and enhancing TMZ sensitivity by combining O6-BG and TMZ with appropriate drug delivery vehicles may serve as a promising therapeutic strategy for GBM.

In this context, chemotherapeutic and imaging agent-based nanotechnology represents a new paradigm in cancer diagnostics that aims to provide versatile cargos with excellent biocompatibility, protection through refined pharmacokinetics, and enhanced precision through cellular and molecular targeting, thereby making them an ideal platform for targeting cancer and exploiting them for personalized cancer therapy (Alphandéry 2020; Nehra et al. 2021). To optimize the therapeutic efficacy and minimize the systemic toxicity of TMZ, a large spectrum of drug delivery vehicles, such as liposomes, polymeric nanoparticles, and metal-based nanoparticles, have been widely explored as a strategy against GBM (Fang et al. 2015; Kudarha and Sawant 2021; Qu et al. 2016; Sandbhor et al. 2023). In an earlier study, they showed that TMZ-loaded superparamagnetic iron oxide nanoparticle (SPION)-based polymeric nanoparticles tagged with anti-nestin antibody and transferrin to target glioblastoma effectively increased BBB permeability and contributed to cell death while sparing healthy brain cells (Prabhu et al. 2017). In recent years, combination therapy has been shown to play a promising role in glioma treatment (Liu et al. 2022). Bingyang Shi and colleagues extensively studied nanocarrier-based combination therapy for GBM. They developed ApoE-functionalized artesunate-phosphatidylcholine liposomes loaded with TMZ, which significantly decreased the glioma burden and improved the survival rate in vivo (Ismail et al. 2022a). Another study showed that combining TMZ and cisplatin loaded in glioma cell membrane-camouflaged biomimetic nanoparticles to take advantage of targeted cancer cells resulted in potent anti-glioma effects with no systemic toxicity (Zou et al. 2022). However, only few studies have explored the role of dual drug combination (TMZ & O6-BG) nanoplatforms (NPs) (Ramalho et al. 2019; Zhao et al. 2020) for GBM. This might be because the loading of two different molecules with distinct properties, such as differences in molecular weight and hydrophilicity with controlled physicochemical properties, increases the size of the NPs and reduces the entrapment efficiency, which could compromise the efficacy of the drug-loaded NPs. Among all the nanoparticles explored in glioma therapy, lipid-based nanocarriers, especially liposomes, are widely used. Because of their potential to encapsulate both hydrophilic and hydrophobic molecules, they have been utilized as efficient drug carriers (Hegde et al. 2022). Liposomes readily merge with tumor cells because the lipid bilayer is composed of phospholipids and cholesterol, which are highly similar to the cell membrane. As a result, liposomes are recognized as a safe, biodegradable, and biocompatible mode of drug delivery (Gao et al. 2015). Conventional liposomes are rapidly cleared by the mononuclear phagocyte system (MPS), which is mainly found in the liver and spleen and has a short circulation half-life (Cattel et al. 2003). PEG, a hydrophilic polymer used to PEGylate liposomes, increases the circulation half-life, thereby providing improved activity for a longer duration (Yang et al. 2007). Therefore, we intended to develop PEGylated liposomes loaded with TMZ and O6-BG (T + BG@PEG-LP), which can simultaneously target the GBM. In this study, 1,2-Dipalmitoyl-sn-glycerol-3-phosphocholine (DPPC), Soy L-α-phosphatidylcholine (SPC), and cholesterol were used as carrier materials. The entrapment efficiency and particle size (nm) of the liposomes were optimized. The optimized T + BG@PEG-LP was also assessed for its in vitro drug release and surface morphology. The cellular uptake of liposomes was evaluated in malignant glioma cells. U87 MG glioma cells were used as a model cell line to investigate in vitro cytotoxicity. This approach may serve as a foundation for targeted combinatorial delivery for GBM. The liposomal formulation ensures that both drugs are delivered at optimal ratios, maximizing their synergistic effects and providing a strong basis for further in vivo studies and a platform for clinical trials.

## Materials and methods

O6-BG was purchased from Cayman Chemicals (Michigan, USA), and bovine serum albumin (BSA) and 4% paraformaldehyde were procured from Himedia, India. DPPC was purchased from Avanti Polar lipids (USA). TMZ, cholesterol and SPC were purchased from Sigma‒Aldrich, USA. Ethanol, DMSO, methanol, and all other chemicals and reagents used were of analytical grade.

### Preparation of dual drug-loaded liposomes

TMZ and O6-BG-loaded liposomes were formulated via the thin film hydration method, as reported in previous studies, with slight modifications (Aithal et al. 2011). Owing to the lipophilic nature of TMZ, it is loaded into the lipid bilayer of liposomes rather than the aqueous core. The lipophilic nature of TMZ makes it compatible with the lipid membrane of liposomes, facilitating its incorporation into the lipid layer during the formulation process. The lipids included SPC, DPPC, cholesterol, and DSPE-PEG_2000_ (7:2:0.9:0.1 molar ratio)_._were accurately weighed and dissolved in a mixture of chloroform/methanol (8:2 v/v) in a round bottom flask. This step involved gentle agitation to ensure uniform distribution and dissolution of the lipid components. Once the lipids were completely dissolved and evenly dispersed in the solvent mixture, pre-dissolved TMZ and O6-BG at a weight ratio of 1:2 (TMZ: O6-BG, w/w) were added to the lipid mixture. The solutions of drugs (TMZ & O6-BG) and lipids were subjected to evaporation under vacuum (474 mbar) at 55 °C and 120 rpm until the solvents were completely dry. The resulting lipid layer was hydrated with phosphate buffer (pH 6.5). It has been reported that TMZ undergoes rapid hydrolysis at a physiological pH of 7.4 and is converted into 5-aminoimidazole-4-carboxamide (AIC), which has poor permeation through the BBB (Friedman et al. 2000a; Waghule et al. 2021). TMZ and O6-BG exhibit optimal stability at slightly acidic pH values. Hydrating the lipid layer with a buffer at pH 6.5 ensures that the encapsulated drugs remain stable. Therefore, we chose a hydrating buffer at pH 6.5 for liposome preparation. Furthermore, the prepared formulation was subjected to sizing by sonication in a bath sonicator for 15 min, followed by probe sonication for 5 min with a 10 s on/off pulse cycle. Unentrapped drugs were separated by ultracentrifugation at 25,000 rpm for 45 min (Sorvall WX Ultra Series Centrifuge; Thermo Scientific, Hudson, New Hampshire, USA). The supernatant was collected to quantify the unentrapped drugs. The buffer was used to redisperse the liposomes, which were lyophilized and stored at −20 °C in airtight amber glass vials until further testing**.** For the cell uptake study, coumarin 6 (C6), a lipophilic fluorescent dye, was loaded inside the liposome similar to the above preparation. The unentrapped dye was removed by ultracentrifugation and stored in the dark.

#### Characterization of liposomes

##### Particle size, zeta potential, and PDI

The mean vesicle size, polydispersity index (PDI), and zeta potential of the prepared liposomal formulations were measured via the dynamic light scattering method with a Malvern ZS90 zeta-sizer after 100-fold dilution with distilled water.

##### Morphological assessment

The morphological features of the liposomes were assessed via transmission electron microscopy (TEM). Briefly, after ten-fold dilution with buffer, the liposomal dispersion was applied to carbon-coated copper grids and dried under vacuum pressure. Next, 1% phosphotungstic acid was used to stain the samples, which were directly examined and photographed via a high-resolution transmission electron microscope (JEOL 3010, 300 kV). Furthermore, the shape and size of the liposomes were analyzed by scanning electron microscopy (SEM, ZEISS, EVO-18, Germany). Briefly, lyophilized liposomes were applied to carbon grids and dried under vacuum pressure. The dried liposomes were directly examined and photographed via high-resolution scanning electron microscope.

##### Fourier transform infrared (FTIR) spectroscopy analysis

Infrared spectra of the pure TMZ, O6-BG, lipid, and liposome formulations were recorded on an FTIR spectrometer (Shimadzu, USA) via the KBr disk method from 4000 to 500 cm^−1^.

### Entrapment efficiency and drug loading estimation

The entrapment efficiency of O6-BG and TMZ in liposomes was quantified via reverse-phase high-performance liquid chromatography (RP-HPLC) (Waters Alliance 2695, USA) via a C18 column (Eclipse XDB-C18 column; 4.6 × 250 mm, 5 μm; Agilent Technologies, USA) and a UV detector at λ_max_ 235 nm for O6-BG and 329 nm for TMZ. The  amount of O6-BG and TMZ in the supernatant were quantified. For the estimation of O6-BG, the column was eluted in isocratic mode using HPLC-grade methanol (Sigma‒Aldrich, USA) and 0.05 M sodium dihydrogen phosphate buffer (pH 7.0) mobile phase at a ratio of 55:45 with a flow rate of 1 ml/min (Qian et al. 2013). The TMZ was estimated under similar conditions using acetonitrile and 0.2% acetic acid at a ratio of 90:10. To check drug loading, the lyophilized liposome powder was subjected to lysis by using a 1% Triton-X and methanol mixture and sonication for 15 min to complete the release of the drugs, after which it was centrifuged at 12,000 rpm for 10 min at 4 °C. The resulting supernatant containing the drugs was diluted with the mobile phase and measured via HPLC. The amounts of BG and TMZ encapsulated in the LPs were subsequently estimated as follows:

$${\text{ Encapsulation efficiency }}\left( {\text{\%}} \right) = { }\frac{{{\text{Total drug}} - {\text{Unentrapped drug}}}}{{\text{Total drug}}}{ } \times 100,$$

$${\text{Drug loading }}\left( {{\text{DL}}} \right){\text{\% }} = \frac{{\text{Drug in nanoparticle}}}{{\text{Total weight of the nanoparticle}}} \times 100.$$

### In vitro drug release

The in vitro release of O6-BG and TMZ from liposomes was analyzed via a dialysis method as previously reported, with minor modifications at different pH values (Ismail et al. 2022a). Briefly, pure drugs and the liposomal dispersion containing 2 mg of drugs were placed in separate dialysis bags (MWCO: 12,000 Da, Sigma–Aldrich Co., Missouri, US) ™. The dialysis bags were immersed in 40 ml of phosphate buffer (pH 5.5 and pH 6.8) and PBS (pH 7.4) and kept on a magnetic stirrer with constant stirring at 37 °C. At predetermined time intervals (0–72 h), 1 ml of release media was collected, replaced with the same volume of fresh buffer and centrifuged at 10,000 rpm for 10 min, and the supernatants containing the released drugs were collected for HPLC analysis. We used an earlier reported analytical method to quantify intact TMZ (Afzalipour et al. 2019; Zou et al. 2022).

### Stability studies

#### Temperature stability

The stability of both plain and drug-loaded liposomes was investigated for 50 days. For temperature stability studies, we kept both plain and drug-loaded liposomes at room temperature (25 ± 2.0 °C) with 32 ± 2.0% relative humidity, and the other set of formulations was kept in a refrigerator (4.0 ± 2.0 °C). At regular intervals, the physical characterization of the liposome suspension was evaluated by measuring its size, PDI, and zeta potential (Kudarha and Sawant 2021).

#### pH stability

The influence of pH on liposome stability and size was determined via previous methods with minor modifications (Kudarha et al. 2015). The lyophilized powder was dispersed in PBS at different pH values (4.0, 6.0, and 7.4) and incubated for 24 h at 37 ± 0.5 °C. The physicochemical characteristics were analyzed at fixed time intervals by measuring the particle size and PDI.

### Biocompatibility assays of liposomes

#### Interaction with serum protein and erythrocyte

The impact of serum proteins on the stability of T + BG@PEG-LP was investigated through protein adsorption experiments. Protein adsorption onto the surface of nanoparticles can significantly influence their stability, biodistribution, and overall therapeutic efficacy, as noted in various studies on nanoparticle‒protein interactions. Human serum albumin (HSA) was utilized as a model protein in this work because of its abundance in blood plasma (Thakur et al. 2014). In this study, 10 mg of lyophilized T + BG@PEG-LP powder was reconstituted in PBS at pH 7.4 and then mixed with a 40 mg/mL HSA solution. The concentration of HSA was chosen on the basis of the plasma concentration (Merlot et al. 2014). The resulting mixture was placed on an orbital shaker set to 100 rpm and maintained at 37 ± 0.5 °C for 2 h to simulate body temperature. Samples were taken at various time intervals to monitor interactions over time. These samples were centrifuged to remove any unbound or loosely bound proteins, ensuring that subsequent measurements reflected only the stable interactions between HSA and the T + BG@PEG-LPs. The stability of the liposomes postinteraction with HSA was assessed by measuring changes in size, zeta potential, and polydispersity index (PDI). T + BG@PEG-LPs were evaluated for their ability to interact with erythrocytes (RBC) to test the hemocompatibility of the nanoparticles (Sawant et al. 2016). In short, blood was collected from the mouse via a retro-orbital plexus puncture upon ethical considerations, and it was spun for 10 min in tubes containing an EDTA solution. The erythrocyte pellets were washed three times with PBS. The drug mixture and liposomes were added to the erythrocyte solution and incubated at 37 °C for 2 h. Erythrocytes in saline were used as the control group. Triton X-100 treated erythrocytes have been kept as a positive control for hemolysis. A bright-field microscope was used to view the alterations in RBC morphology by nanoparticles.

## Malignant glioma cell lines

The U87 MG cells were maintained in DMEM containing 10% FBS and antibiotic solution at 37 °C and supplied with 5% CO_2_. Early-passage cells were used in all the experiments.

### Estimation of cellular uptake

The cellular uptake of liposomes in U87 cells was assessed via confocal laser scanning microscopy (CLSM) according to previous methods, with slight modifications (Liu et al. 2021). Both plain fluorescent dye and coumarin-6 (C6) dye-loaded LPs have been employed. Briefly, cells (2 × 10^5^ cells) were grown on coverslips coated with 0.1% gelatin. Once the cells were attached, C6 dye-loaded PEGylated LPs in serum-free media (0.1 mg/mL C6 equivalent) and coumarin-6 alone as a control were added and incubated for 4 h. The cells were subsequently washed twice with ice-cold PBS and fixed with 4% paraformaldehyde, and 4′,6-diamidino-2-phenylindole (DAPI) (0.5 µg/ml) was used to stain the nucleus, which was subsequently mounted on a glass slide with anti-fading mounting medium. The images were captured via CLSM at 63x and higher magnification at 100x (excitation wavelength, 428 nm; emission wavelength, 512 nm). The quantitative estimation of the cellular uptake of the C6-loaded liposomes compared with that of the free dye was performed via flow cytometry. Initially, 2,00,000 U87 MG cells were seeded into 6-well plates and allowed to adhere overnight. On the following day, the culture medium was replaced with serum-free DMEM containing 0.1 mg/ml free coumarin 6 dye, and an equivalent amount of dye encapsulated within liposomes (C6@PEG-LP) was added to separate wells. The cells were then incubated for 4 h at 37 °C in a humidified incubator with 5% CO_2_ to facilitate dye uptake. After the incubation period, the medium was removed, and the cells were washed twice with PBS to remove any noninternalized or excess dye adhering to the cell surface. Trypsinization was performed to detach the cells from the wells, and the cells were collected. The degree of internalization of the coumarin 6 dye was quantified by measuring the fluorescence intensity of the cells via a flow cytometer (Partec, FACS Calibur, GmbH, Munster, Germany).

### In vitro cytotoxicity

Cytotoxicity assessment of pure TMZ and O6-BG (1–200 µg/mL) on U-87MG cells was performed via the MTT assay, and the inhibitory concentration to kill 50% of the cells (IC_50_) was calculated. After calculating the IC_50_ of the pure drugs, we assessed the cytotoxic effect of our nanoformulation compared with that of the pure drug combination. The cells were grown in 96-well plates (5 × 10^3^ cells/well) and were incubated overnight until they attached. After 24 h of culture, the cells were subjected to various treatment regimens. These included groups treated with various concentrations of TMZ (TMZ) alone (1 to 50 µg/mL), a combination of TMZ and O6-BG at concentrations ranging from 1 to 50 µg/mL, and 1 to 100 µg/mL, respectively, and a group treated with PEGylated liposomes (T + BG@PEG-LP) loaded with TMZ at concentrations ranging from 1 to 50 µg/mL equivalent and O6-BG at concentrations equivalent to 1 to 100 µg/mL. The cells were subsequently incubated for 72 h to allow for drug uptake and cytotoxic effects. After treatment, the drug-containing media was discarded, and 100 µl of MTT solution from a 1 mg/mL stock mixture was added to each well and further incubated for 4 h. Following incubation, the formed formazan crystals were dissolved by adding 100 µl of dimethyl sulfoxide (DMSO)/well, and the colorimetric absorbance was measured at a wavelength of 570 nm (Das et al. 2017). The combination index of TMZ and O6-BG cotreatment was calculated via the Chou‒Talalay method (Chou 2010).

#### Determination of apoptosis via annexin V-FITC double-staining

To determine the effects of TMZ and TMZ + O6-BG in contrast to T + BG@PEG-LP in inducing cell death via apoptosis, the effects were assessed via the PI/Annexin V-FITC double-staining method as described previously (Das et al. 2017) and with a kit supplier (Sigma Aldrich, USA). Exponentially growing U87 cells were seeded (2.5 × 10^5^ cells) and treated with a higher dose of TMZ (20 µg/mL), a lower dose of TMZ + BG (5 µg/mL or 10 µg/mL), or T + BG@PEG-LP (5 µg/mL or 10 µg/mL) for 72 h. After various treatments, the cells were trypsinized and washed with sterile PBS. The cells were suspended in 1X binding buffer and stained in the dark with fluorescein isothiocyanate (FITC)-labeled Annexin V (5 μl) and propidium iodide (PI) (10 μl) for 5 min, followed by analysis via a flow cytometer (Partec, FACS Calibur GmbH, Munster, Germany).

### Cell cycle analysis via propidium iodide (PI) staining

Cell cycle analysis was performed according to previous methods (Das et al. 2017). The cells were seeded at a density of 2.5 × 10^5^ cells per 6-cm plate and allowed to grow overnight. The cells were treated with TMZ (20 µg/mL), TMZ + BG (5 µg/mL or 10 µg/mL), or T + BG@PEG-LP (5 µg/mL or 10 µg/mL) and incubated for 72 h. After incubation, the cells were trypsinized, and the cell pellets were obtained by centrifuging at 1000 rpm for 10 min. Following washing with PBS, the cells were fixed in 70% cold ethanol, followed by incubation at 4 °C for 15 min. The samples were then centrifuged, the fixed cells were rinsed twice with PBS, and RNase A (100 µg/mL) was added, followed by incubation at 37 °C in a water bath for an hour. Next, 10 µL of propidium iodide (1 mg/ml stock) was added to the cells and incubated at 4 °C for 30 min. The cells were then resuspended in PBS, and flow cytometric analysis was performed. The results were analyzed via WinMDI software.

## Statistical analysis

All the data were analyzed in triplicate and are presented as the mean ± SD. The statistical significance was calculated via an unpaired t test or one-way ANOVA with GraphPad InStat software (La Jolla, CA, USA). A *p* value of < 0.05 was considered to indicate statistical significance.

## Results and discussion

The DNA damage-inducing alkylating drug TMZ and radiotherapy are the current standards of care for treating high-grade gliomas (GBMs) (Friedman et al. 2000a, b). TMZ is extensively used in clinical settings to treat GBM; however, chemotherapeutics in general, particularly TMZ, have systemic absorption, resulting in bone marrow suppression and leading to transient lymphopenia and thrombocytopenia, which affects treatment compliance and ultimately compromises clinical outcomes. The synergistic combination of drugs has the potential to decrease drug resistance while minimizing the required cytotoxic dose (Ghosh et al. 2018). Nevertheless, the increased efficacy of combination drugs is hindered by added associated toxicities. High dosages are often used to reach a therapeutic drug level, which results in notable off-target toxicity (Bhatia et al. 2020; Lopez and Banerji 2017). In numerous preclinical and clinical studies, TMZ has been combined with other drugs to enhance its therapeutic efficacy against glioblastoma (Dhungel et al. 2024; Graham-Gurysh et al. 2020; F.C. Lam et al. 2018a; Nieder et al. 2006). The combination therapy of TMZ and O6-BG is based on the principle of overcoming chemoresistance mediated by MGMT. When used together, O6-BG sensitizes tumor cells to TMZ by inhibiting MGMT, increasing DNA damage accumulation. Despite the promising synergistic effects, there are several limitations to the combination therapy of TMZ and O6-BG. Combining O6-BG with TMZ can lead to increased toxicity. O6-BG, however, also lowers MGMT levels in normal cells, which makes chemotherapy more toxic and increases the risk of brain infections, bone marrow suppression, and hydrocephalus (Quinn et al. 2009a; Schold et al. 2004).

In the present study, we went a step ahead and hypothesized that the inclusion of O6-BG with TMZ in a nanoformulation would increase the susceptibility of high-grade gliomas to TMZ treatment. Owing to their distinct physicochemical characteristics, such as stability, biocompatibility, and biodegradability, liposomes have attracted increasing interest as drug delivery methods for GBM in recent years (Gao et al. 2015; Lin et al. 2018; Torchilin 2005). The primary aim of the current investigation was to construct a dual drug-loaded T+BG@PEGLP and examine its anti-cancer effectiveness against high-grade gliomas such as GBM under in vitro conditions.

### Physicochemical properties of T + BG@PEG-LP

**Fig. 1:**
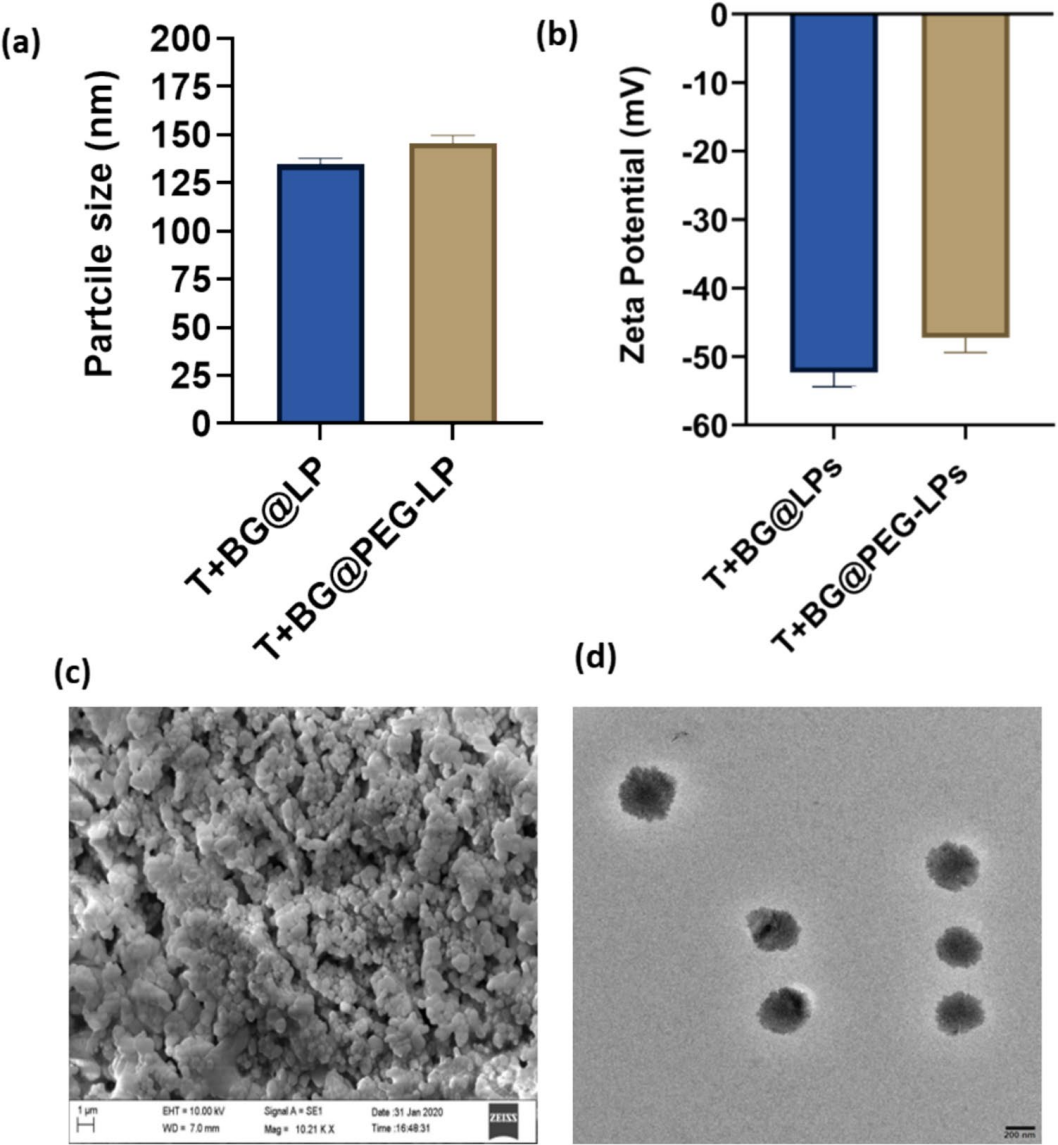
Physicochemical characterization of T + BG@LP and T + BG@PEG-LP liposomes: **a** bar graph representing the particle size with an average particle size of 146.33 ± 6.75; **b** zeta potential showing the slight decrease after PEGylation; **c** representative SEM image; and **d** TEM image of T + BG@PEG-LP depicting a spherical vesicle shape with homogeneous dispersion (n = 3)

The thin-layer hydration technique was used to develop T+ O6-BG@PEG-LPs by allowing SPC, cholesterol, DPPC, and DSPE-PEG_2000_ to self-assemble. SPC has a fluidizing effect on lipid packing, is naturally produced, is biocompatible, and improves cellular absorption (Le et al. 2019). The hydrophilic polymer PEG, on the other hand, can be employed to coat nanoparticles or to copolymerize (PEGylate) the lipids utilized to extend the retention life of nanoparticles in circulation. PEGylation imparts a stealth-like property to the liposomal nanocarrier by forming a hydrophilic layer around its surface. This stealth effect reduces recognition and clearance by the reticuloendothelial system (RES) and prolongs the circulation time in the bloodstream (Gajbhiye et al. 2020; Mozar and Chowdhury 2018). As a result, PEGylated liposomes can evade systemic clearance and effectively penetrate biological barriers, including the BBB. This facilitates the translocation of liposomal nanocarriers across the BBB via various mechanisms, including receptor-mediated transcytosis and adsorptive-mediated transcytosis (Johnsen et al. 2019; F.C. Lam et al. 2018a). PEGylation reduces the immunogenicity of liposomal nanocarriers, thereby minimizing immune recognition and clearance (Gajbhiye et al. 2020; Hu et al. 2019). This property is particularly advantageous for brain targeting, as it ensures prolonged circulation and sustained drug release within the brain microenvironment, leading to enhanced therapeutic efficacy against glioblastoma. Numerous studies have investigated the efficacy of PEGylated liposomes for treating glioblastoma, highlighting their potential as promising drug delivery systems for targeted therapy (Gajbhiye et al. 2020; Hu et al. 2019; Sandbhor et al. 2023). A recent study by Ghaferi et al. reported the anticancer efficacy of doxorubicin and carboplatin-loaded PEGylated liposomes in treating glioblastoma. Compared with that of plain liposomes, the survival rate of glioma-bearing rats significantly increased by 10% when they were treated with PEG-Lip-DOX/CB (Ghaferi et al. 2022). The added efficacy of dual drug combinations, as shown by various groups, sets the premise for the present study for combining two effective anti-glioma drugs for targeted therapy via nanoformulation. As the physicochemical characteristics of the NPs determine their biological fate and toxicity, the resulting nanosystems must satisfy several requirements to be suitable for targeted intracranial delivery into the brain. In this study, the prepared nanoformulation had a particle size < 200 nm and a dispersed homogenous distribution, which has a greater potential to cross the BBB and the BBTB (Betzer et al. 2017). Recent research has demonstrated that liposomes with sizes less than 200 nm exhibit an enhanced ability to traverse the BBB. Lin et al. and colleagues reported that doxorubicin-loaded liposomes with a particle size of 187.02 nm were able to cross the BBB and showed enhanced tumor regression in a mouse model (Lin et al. 2016). In another study, TMZ- and quercetin-loaded PEGylated liposomes had an average particle size of 196.5 ± 47.3 and had a greater brain distribution than other types of liposomes did, which was attributed to the brain uptake of the nanoliposomes (Hu et al. 2016). In support of the findings of the above study, TMZ-loaded liposomes targeting glioblastoma were relatively uniform in size (156.70 ± 11.40) (Gao et al. 2015). Indeed, incorporating multiple drugs into liposomes can pose challenges in reducing the particle size. When two drugs are loaded into liposomes, maintaining a small particle size can be challenging because of the increased payload and potential interactions between the drugs and the lipids. Initially, the liposome formulation was optimized by altering the amount of different excipients and drugs with different molar ratios of the lipids. The optimized liposomes with SPC, DPPC, cholesterol, and DSPE-PEG_2000_ at a molar ratio of 7:2:0.9:0.1 were chosen. TMZ is a lipophilic drug (Barciszewska et al. 2015; Yin et al. 2023) that is typically incorporated into the lipid bilayer rather than the aqueous core of liposomes. The lipophilic nature of TMZ allows it to integrate with the lipid membrane during the formulation process. It is well-documented in the literature that TMZ is chemically unstable at physiological pH (7.4) due to its hydrolytic degradation into the active methylating agent (Beale et al. 1999; Stéphanou and Ballesta 2019). TMZ and O6-BG are known to have different solubility properties. TMZ is moderately soluble in water. On the other hand, O6-BG is much less water-soluble and requires organic solvents for effective dissolution (Ramalho et al. 2019). The solubility profile of TMZ and O6-BG was addressed through their encapsulation in liposomes, which enhanced the overall solubility and bioavailability of both drugs. The physicochemical properties of the prepared formulations were characterized via dynamic light scattering (DLS), zeta sizer, and the details are presented in Table 1. The plain liposomes had a mean particle size of 132.24 ± 5.32 nm (Fig. 1a). However, adding PEG increased the size to 146.33 ± 6.75 nm (Fig. 1b). The observed PDI was less than 0.35. The size of our PEGylated liposomes was approximately 150 nm, which falls within the optimal range (100–200 nm) for enhanced permeability and retention (EPR) effect. In comparison, non-PEGylated control liposomes exhibited smaller sizes, which may either reduce tumor accumulation or lead to premature clearance, limiting therapeutic efficacy. In a recent study, non-PEGylated liposomes showed lower tumor uptake and shorter circulation times compared to PEGylated formulations (Waghule et al. 2023). The zeta potential of our liposomes was −49.6 ± 3.1 mV, indicating a negative charge, which is favorable for systemic circulation. Neutral or slightly negatively charged liposomes are known to have reduced opsonization and slower clearance by macrophages compared to highly positively charged liposomes (Chauhan et al. 2020). The entrapment efficiency of both drugs was similar in both nanoformulations, ranging from 36.3 ± 4.1% to 42 ± 6.0% for TMZ and 69.4 ± 2.4% to 72.4 ± 3.6% for O6-BG. The drug loading ratio of TMZ to O6-BG was maintained at 1:2 (w/w), which is approximately equivalent to the dose ratio given in clinical trials (Quinn et al. 2009b). In an earlier study, the loading efficiency was 3.05% for BCNU and 8.97% for O6-BG (Qian et al. 2013), which also corroborates the findings of the present study. This superior loading efficiency of the liposomes would be highly beneficial, as it would reduce the need for excipients, which would not only reduce collateral adverse effects due to normal tissue damage but also enhance treatment efficacy and improve patient compliance, thereby improving the therapeutic ratio of the given strategy. Scanning electron microscopy (SEM) (Fig. 1c) and transmission electron microscopy (TEM) (Fig. 1d) and revealed homogenous vesicles and no aggregation between particles. Generally, the particle size measured by DLS is greater than that measured by TEM or SEM, which may be due to the hydrodynamic diameter of the particles in DLS (Fissan et al. 2014; Maguire et al. 2018). However, in the present study, the particle size determined via TEM/SEM was greater, although negligible, than that determined via DLS, which may be due to the differences in the principles and limitations of these methods. SEM/TEM provides high-resolution images of particles, but the measured size can be influenced by factors such as charging effects and the blurring of particle edges, especially for smaller particles, leading to larger apparent sizes.

**Table 1:** Physicochemical characterization of liposomes

Nanocarrier	Size (nm)	Zeta potential (mV)	PDI	Entrapment efficiency (%)	Drug loading (%)
T + BG@LPs	132.24 ± 5.32	−52.1 ± 2.8	0.223 ± 0.01	TMZ: 36.3 ± 4.1 O6-BG: 69.4 ± 2.4	3.8 ± 1.4 5.6 ± 2.2
T + BG@PEG-LPs	146.33 ± 6.75	−49.6 ± 3.1	0.328 ± 0.03	TMZ: 42 ± 6.0 O6-BG:72.4 ± 3.6	3.5 ± 1.3 6.8 ± 2.0

The results are presented as the mean ± SD, n = 3

#### Fourier transform infrared (FTIR) spectroscopy analysis

**Fig. 2:**
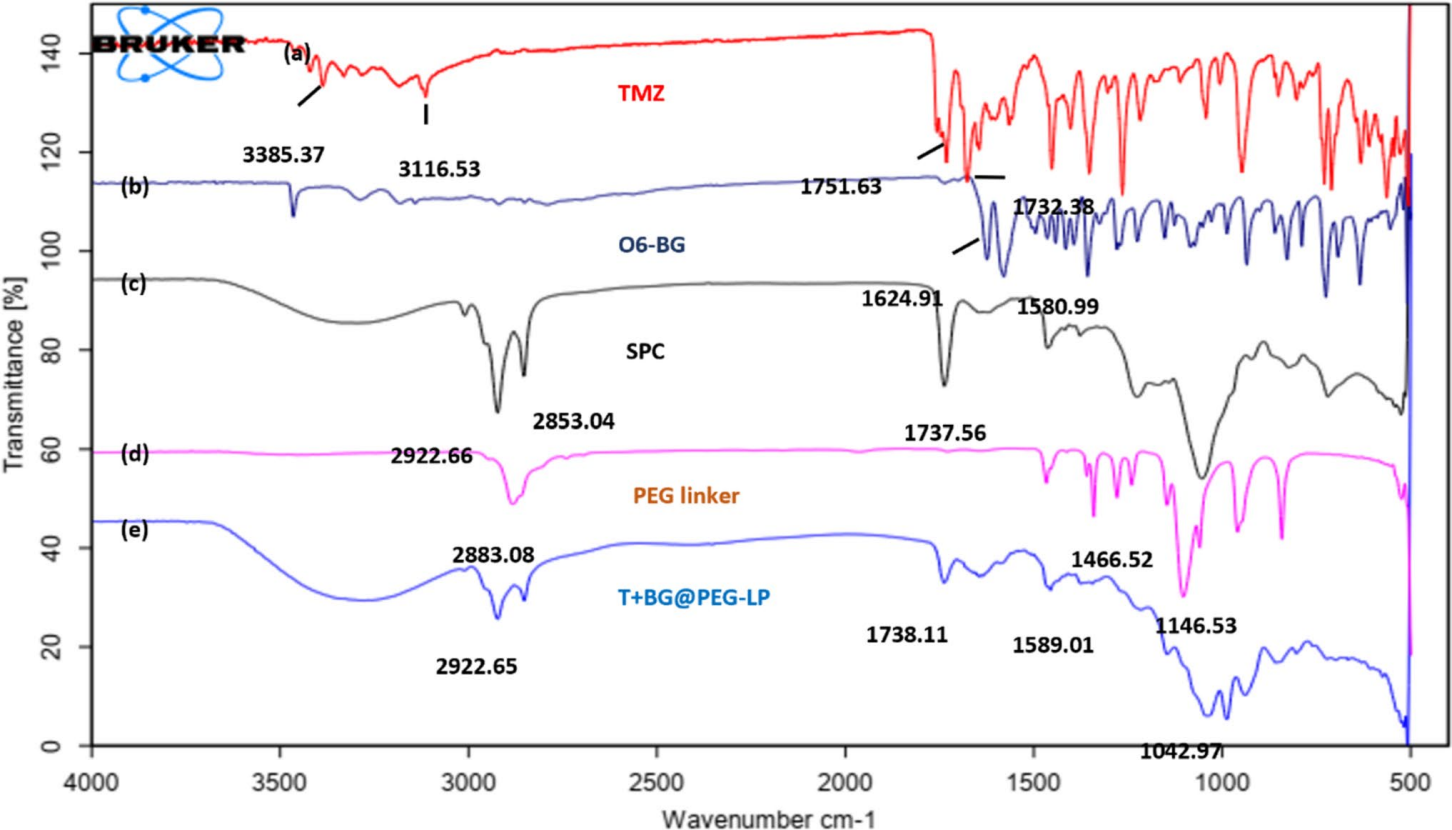
Overlay of the IR spectra of **a** TMZ **b** O6-BG **c** SPC **d** DSPE-PEG_2000_ **e** T + BG@PEG-LP. Spectra of TMZ showing three broad bands at 3387, 1743.65 and 1680 cm^−1^ given by the stretching vibration modes of NH2, C=O and OH– groups. In O6-Benzylguanine spectra, 2 sharp peaks were observed at 1633.71 cm^−1^ and 1583.56 cm^−1^ given by C=N and benzene ring, respectively

The pre-formulation study results are provided in the supplementary file S1. The overlays of the IR spectra of TMZ, SPC, DSPE-PEG_2000,_ and PEG-liposomes are shown in Fig. 2. The FTIR spectrum of TMZ displayed an absorption band at 3385.37 cm^−1^ due to the presence of NH stretching. The band at 3116.53 cm^−1^ was due to the asymmetric stretching of alkyl groups. The stretching vibrations at 1732.38 cm^−1^ and 1751.63 cm^−1^ correspond to the primary and tertiary amides of TMZ, respectively (Wang et al. 2021). In the O6-BG spectra, 2 sharp peaks attributed to the C=N and benzene rings were observed at 1633.71 cm^−1^ and 1583.56 cm^−1^, respectively. Furthermore, the FTIR spectrum of SPC showed characteristic absorption peaks at wavenumbers of 2922.66 cm^−1^ and 2853.04 cm^−1^ and at 1737.56 cm^−1^, corresponding to the C–H and C=O vibrational stretches of the two long fatty acid tails present in SPC (Perez-Ruiz et al. 2018; Qayyum et al. 2023). In the spectrum of DSPE-PEG2000, the C-O peak was observed at approximately 1146.53 cm^−1^. Other peaks at 2883.07 cm^−1^ and 1466.52 cm^−1^ correspond to the bending and stretching peaks of methylene, respectively. However, in the spectrum of T + BG@PEG-LP, owing to the presence of lipids, the shape and characteristic peak intensity of TMZ were reduced, indicating the encapsulation of the drug into the liposomes. Furthermore, some representative peaks’ broadening, shifting, or vanishing demonstrates T + BG@PEG-LP formation. The compatibility/stability of the drugs and excipients used in the formulation was assessed by differential scanning calorimetry (DSC) (Fig. S1) and FTIR (Fig. S2).

### Stability studies

#### Temperature and pH effects

**Fig. 3:**
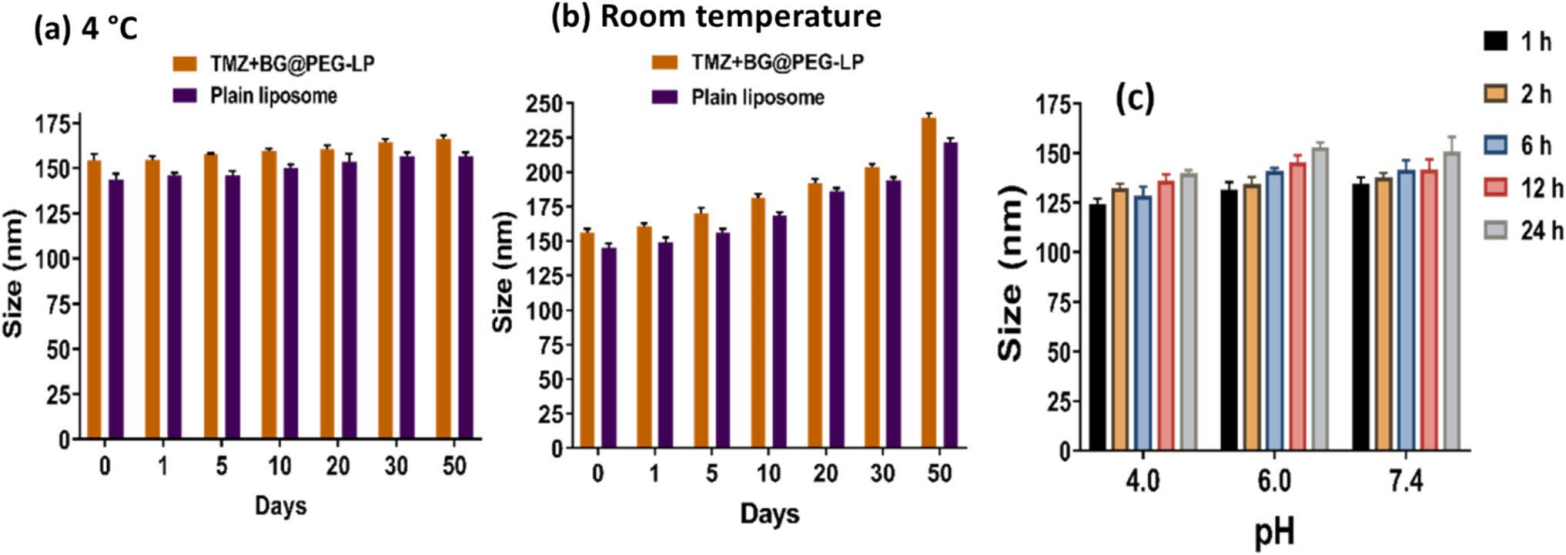
Effects of temperature and pH on liposome stability; **a** LPs and drug-loaded LPs stored at 4 °C are stable with minimum changes in their particle size; **b** LPs and drug-loaded LPs at room temperature (25 ± 2.0 °C) showing the increase in size over the period of time; and **c** the stability of liposomes at different pH values at different time intervals, indicating that the liposomes are stable at physiological pH conditions without any changes in size. The data are presented as the mean ± SD, n = 3

The stability of liposomal formulations strongly depends on the storage temperature. We kept both plain liposomes and T + BG@PEG-LP at 4 °C and at room temperature for 50 days. The liposomes started to aggregate at room temperature over the entire time, as indicated by the increase in size (Fig. 3a). However, liposomes that were stored at 4 °C were stable, with a minimal increase in size. (Fig. 3b). We then analyzed the effects of different pH conditions on the stability of the liposomal formulations. When the liposomes were incubated at different pH values (pH values of 4.0, 6.0, and 7.4) for 24 h, insignificant changes in size were observed up to 24 h, confirming the stability of the liposomes under different physiological conditions (Fig. 3c).

### Biocompatibility studies

#### Interactions with serum proteins and erythrocytes

**Fig. 4:**
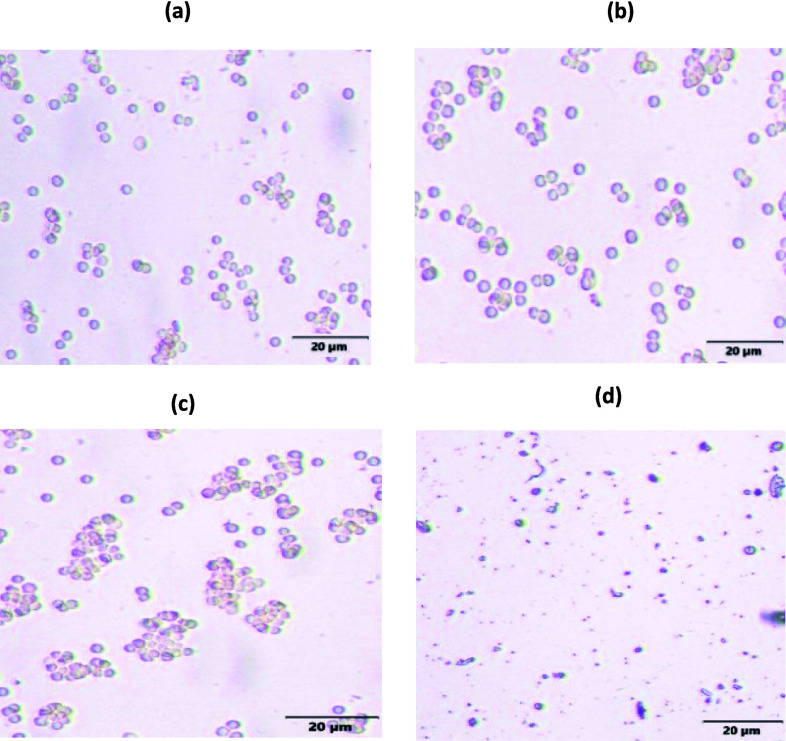
Hemocompatibility study of T + BG@PEG-LP. Representative microscopic image of erythrocytes treated with **a** saline as control, **b** pure drugs, **c** drug-loaded LPs, and **d** Triton X-100 as a positive control. Incubation with pure drugs and drug-loaded liposomes did not cause any disruption/changes in the morphology of the erythrocyte, giving evidence that the tested drugs and liposomal delivery systems are unlikely to induce hemolysis or other ensuring their safety

Several in vitro experiments have been performed to assess the stability and toxicity of liposomes. The binding of liposomes to serum proteins, which changes their surface charge, is a significant impediment to using liposomes for brain administration. Therefore, the stability of empty and drug-loaded liposomes was examined in the presence of human serum albumin (HSA) at a physiological concentration (40 mg/ml) (Merlot et al. 2014; Thakur et al. 2014). The results of our investigation of serum stability demonstrated that the liposomes would be stable within the body since there is little/nonsignificant interaction with the serum, which was supported by the particle size and PDI measurements of the dispersion (Table 2). Because of the small size and distinctive physicochemical properties, parental administration of nanoparticles may interact with erythrocytes, which may result in the hemolysis of red blood cells (de la Harpe et al. 2019). As illustrated in Fig. 4, hemocompatibility studies were carried out to determine the toxicity potential and biocompatibility of the formulation. The treatment with nanoformulations may alter the surface characteristics of RBC, which is not a desirable feature because it could have a detrimental effect on its circulation within the body fluids. RBC morphology was examined under a microscope. We observed that the free drug solution and T + BG@PEG-LP led to no shrinkage or rupture. The results of the above study justified the safety and biocompatibility of the T + BG@PEG-LP formulation. Because of the similar charges between RBC and NP, demonstrating the repulsive effect. This may co-relate to the safety of nanoparticles in future in vivo studies.

**Table 2:** Physical stability of T + BG-LP and T + BG@PEG-LP in human serum albumin (HSA) presence

T + BG-LP			T + BG@PEG-LP	
Time (min)	Size (nm)	PDI	Size (nm)	PDI
0	149.2 ± 2.0	0.348 ± 0.02	191.2 ± 1.0	0.411 ± 0.02
30	153.1 ± 3.1	0.412 ± 0.01	190.3 ± 3.4	0.393 ± 0.08
60	161.3 ± 5.2	0.422 ± 0.12	192.2 ± 2.1	0.455 ± 0.01
120	173.0 ± 1.2	0.398 ± 0.05	198.2 ± 1.9	0.472 ± 0.14

The results are presented as the mean ± SD, n = 3

### In vitro drug release study

**Fig. 5:**
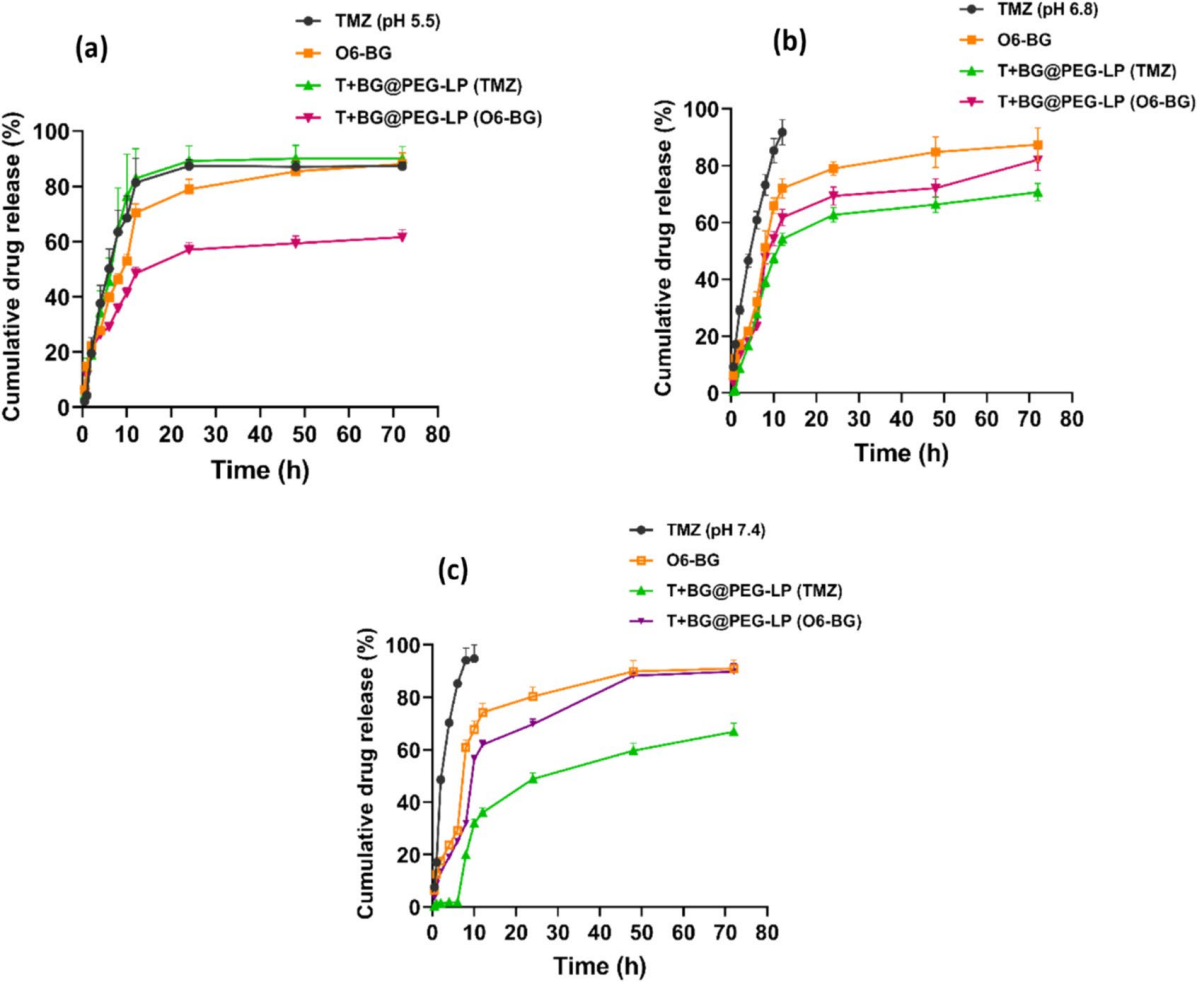
In vitro drug release of TMZ and O6-BG from T + BG@PEG-LP at different pH values: **a** pH 5.5, **b** pH 6.8, and **c** pH 7.4 at 37 °C for 72 h. The release study was carried out via the dialysis method. TMZ was unstable at physiological pH. However, a sustained drug release was observed when the drugs were encapsulated in liposome with a release of 69.70 ± 2.88% at 24 h incubation. The data are presented as the mean ± SD

To determine the release patterns of TMZ and O6-BG embedded in liposomes, we carried out an in vitro release study of both the pure drugs and the nanoformulations. The release profiles of TMZ and O6-BG from T + BG@PEG-LP at different pH values are depicted in Fig. 5. The cumulative drug release of TMZ and O6-BG at pH 5.5 reached 89.98 ± 6.85% and 57.25 ± 3.66%, respectively (Fig. 5a); at pH 6.8, it was 64.57 ± 6.37% and 69.98 ± 4.49%; and at pH 7.4, the release was 66.84 ± 4.62% and 69.70 ± 2.88%, respectively, over a 24 h course. In contrast, an accelerated burst release (~ 95%) of TMZ from the TMZ solution was observed at pH 7.4 as well as at pH 6.8 within 10 h of incubation (Fig. 5b and c), which was similar to earlier reports (Afzalipour et al. 2019; Fred C. Lam et al. 2018a), where they studied the TMZ release behavior at pH 7.4 for up to 72 h. To support our observation, in a recent study by Ismail et al. (2022a), the release of TMZ was significantly faster at acidic pH (pH 5.0) compared to physiological pH (pH 7.4). After 48 h of incubation, approximately 85% of TMZ was released at pH 5.0, whereas a slower release was observed at pH 7.4. This accelerated release in acidic environments can be attributed to acid hydrolysis, which leads to the degradation of the liposomal membrane, facilitating the rapid release of the encapsulated drug. Furthermore, our findings are corroborated by another study by Xu et al. (2021), in which arsenic trioxide (ATO)-loaded liposomes were employed for glioblastoma treatment. The release profile of ATO was evaluated at varying pH levels, specifically pH 7.4, 6.5, and 5.5, mimicking physiological and tumor microenvironment conditions. The study demonstrated a pH-dependent release of the liposomal ATO, with 77.9 ± 4.55% drug release observed at pH 5.5 after a specific period, compared to 57 ± 4.20% release at pH 7.4. This pattern of increased drug release under acidic conditions is consistent with our observations and highlights the potential of liposomal formulations to achieve controlled release based on environmental pH (Xu et al. 2021), findings from both studies provide evidence that liposomal drug formulations exhibit enhanced drug release in acidic conditions due to the structural destabilization of the liposomal membrane. Compared with the fast-release pattern of free drug solutions, the liposomal formulations displayed sustained and slow drug release, which was observed and is anticipated to show prominent therapeutic efficiency.

### Cellular uptake study

**Fig. 6:**
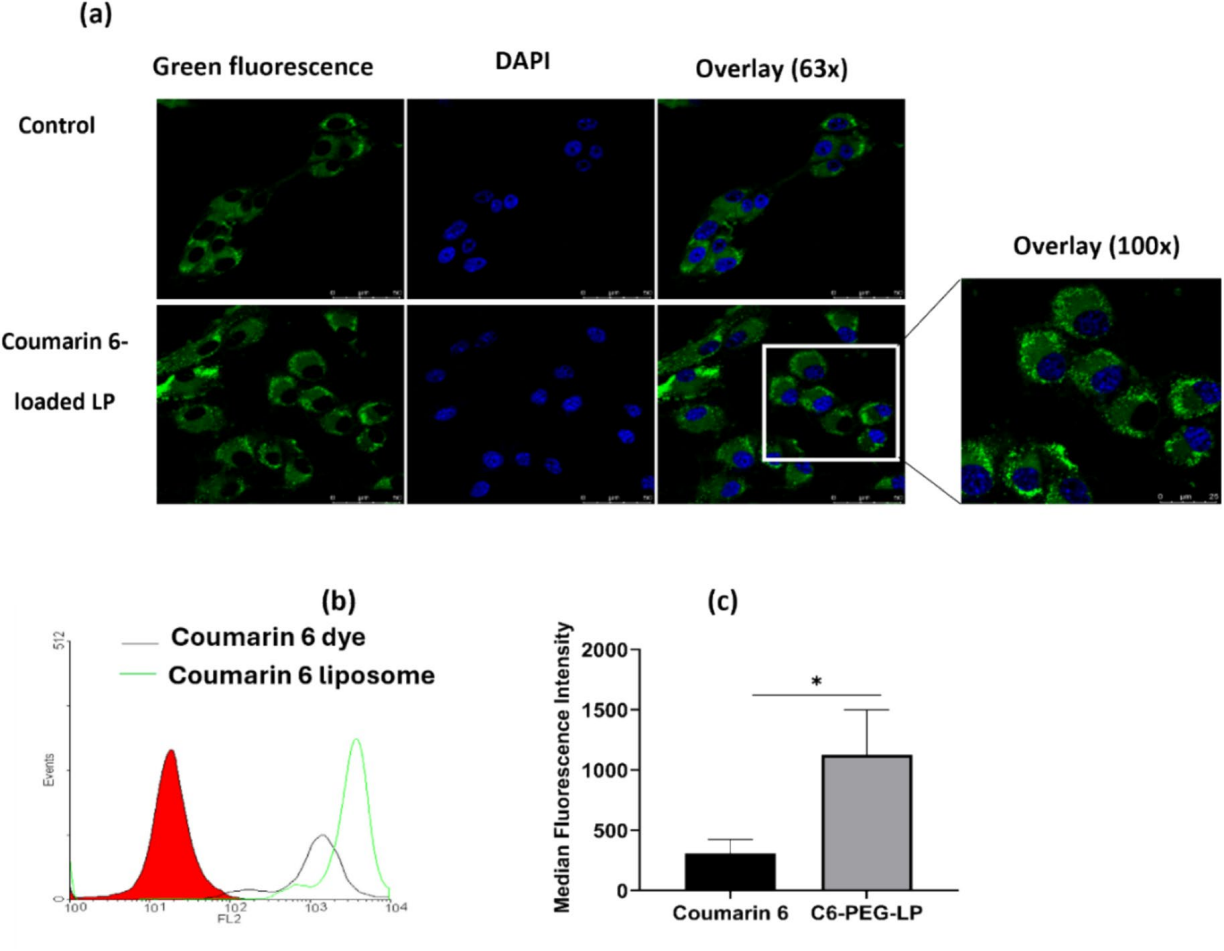
Cellular uptake of coumarin 6- and coumarin 6-loaded liposomes in glioblastoma cells. **a** Representative images of confocal laser scanning microscopy showing the bright intensity of green fluorescence in PEGylated liposome treated cells indicating the higher accumulation at cytoplasm; **b** representative histogram and **c** quantification of U87 cells incubated with coumarin-6 dye and coumarin-6 liposomes for 4 h, a significant increase in cell uptake was seen due to endocytosis of the liposomal vesicles than in the free dye. Data are presented as the mean ± SD; n = 3; *p < 0.05 compared with the dye control

The effectiveness of cellular uptake is regarded as a crucial tool for determining the potential of a nanoparticulate system for drug delivery. To investigate the qualitative and quantitative cellular uptake of the formulated liposomes, U87 MG cells were treated with coumarin-6 dye as a control, C6-PEG-LP, and harvested after 4 h of incubation, and the fluorescence intensity was observed via confocal microscopy. The fluorescent signals clearly revealed that internalization occurred largely in the cytoplasm of the cells (Fig. 6a). The cellular uptake of liposomes primarily depends on the lipid composition (Mateos-Maroto et al. 2023), surface charge, and vesicle size (Foroozandeh and Aziz 2018). The lipid-based nanocarriers utilize clathrin-mediated endocytosis (Foroozandeh and Aziz 2018; Rivolta et al. 2011). In an earlier study, Temozolomide and Quercitin-loaded PEGylated liposomes were more rapidly taken up by U87 glioma cells than the free drugs indicating the liposomal internalization via endocytosis (Hu et al. 2016). Our liposomal formulation had a diameter of approximately 150 nm and a desirable negative surface charge (–29.6 ± 3.1), contributing to the enhanced cellular uptake in U87 MG cells. These characteristics are known to subsidize enhanced cellular internalization. However, conjugating tumor-specific ligands to the liposomes can further increase the cell uptake efficiency via receptor-mediated endocytosis (Juhairiyah and de Lange 2021; Mojarad-Jabali et al. 2021). The median fluorescence intensity is shown in Fig. 6b and c. Compared with the free dye control, C6-PEG-LP displayed the highest uptake (*p* < 0.05).

### Cytotoxicity analysis by MTT assay

**Fig. 7:**
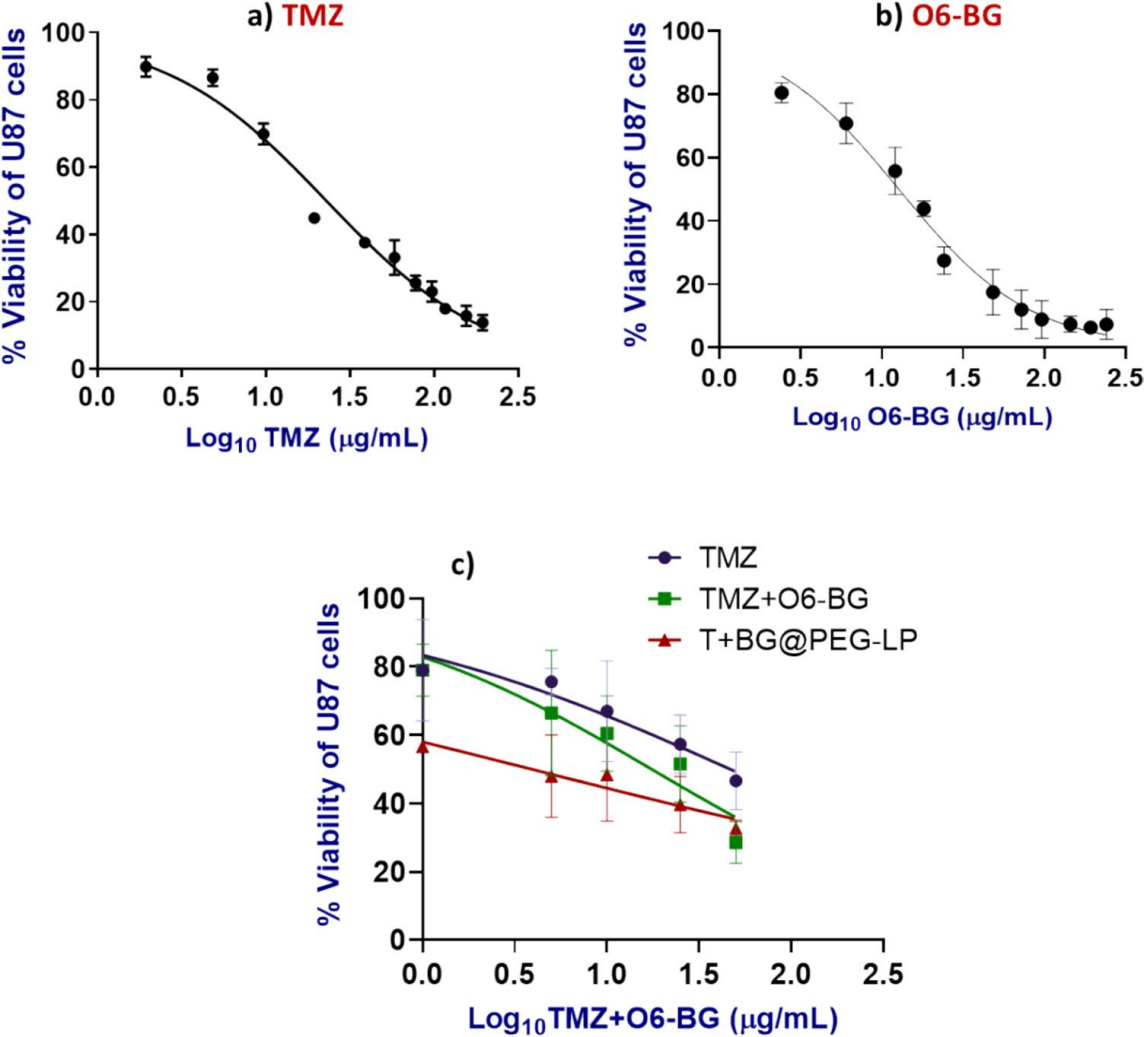
Cell viability of U87 cells after treatment with free **a** TMZ, **b** O6-BG, or **c** a dose-dependent percentage of cell death after treatment with a combination of TMZ + O6-BG or T + BG@PEG-LP. The cytotoxicity was evaluated using the MTT assay after 72 h of incubation. Both the free drug combination and the drug-loaded liposomes exhibited dose-dependent cell death, with a synergistic effect observed in the combination of TMZ and BG. The data are presented as the mean ± SD; n = 3

In our study, our primary aim was to investigate the efficacy and safety of combination drug treatment using TMZ and O6-BG given that O6-BG sensitizes tumor cells to TMZ; therefore, combination therapy would have led to natural translation to the clinic, where drug combinations would be studied in human clinical trials. As such, our experimental design focused on comparing the outcomes of the drug combination in liposomal nanoformulation treatment to those of the groups receiving a combination of plain drugs. The in vitro therapeutic impact of our nanoformulation demonstrated its potential use as a therapeutic modality for GBM. In an earlier study, liposomal TMZ administered via convection-enhanced delivery (CED) failed to demonstrate the advantages of LPs as drug delivery systems (Nordling-David et al. 2017). The effects of the combination of TMZ and O6-BG as free drugs and in the encapsulated form at different concentrations were tested in U87 MG cells. Encapsulating TMZ combined with O6-BG inside the liposome significantly increased the cytotoxicity of the drug to U87MG glioma cells. To assess the cytotoxic effects of pure TMZ and O6-BG, U87 MG cells were treated with different doses (1–200 µg/mL) of TMZ or O6-BG for 72 h. The results demonstrated a dose-dependent decrease in cell viability. A nonlinear regression curve was generated by plotting the concentration (X-axis) against the % viability (Y-axis). The IC_50_ value was 23 µg/mL for TMZ (Fig. 7a) and 12.7 µg/mL for O6-BG (Fig. 7b). The concentration of O6-BG needed to kill at least 50% of the cells was distinctly lower than that needed to kill TMZ, as it was reported that U87 cells are sensitive to O6-BG treatment (Chakravarti et al. 2006). Only a slight improvement in TMZ cytotoxicity was observed after TMZ was combined with O6-BG in its free form. On the basis of the findings of our preliminary studies, the carrier materials did not have any negative impact on cell growth; hence, the cytotoxic effect of encapsulated drugs can be attributed to the increased cytotoxicity of drug-loaded liposomes. Furthermore, the combined effects of both drugs in free form and in nanocarriers were studied. The cells were treated with free TMZ (1–50 µg/ml), TMZ + BG at a ratio of 1:2, or T + BG@PEG-LP containing equivalent concentrations of TMZ and O6-BG for 72 h. Dose-dependent cell death was then observed after 72 h of treatment (Fig. 7c). TMZ tends to hydrolyze in aqueous media at pH 7.4, thus failing to crosslink DNA (Renziehausen et al. 2019). However, enhanced cytotoxicity of TMZ against U87 cells was observed in the T + BG@PEG-LP group. The IC50 value of T + BG@PEG-LP was 3.99 µg/mL. Compared with TMZ, T + BG@PEG-LP reduced cell viability by more than 50% at lower doses. The cell viability decreased to 32.68 ± 2.54 when liposomes were treated with the highest dose of 50 µg/mL TMZ and 100 µg/mL BG, while pure TMZ alone resulted in a viability of 51.12 ± 2.03. The combined effects of free O6-BG and TMZ were analyzed by calculating the combination index (C.I.). The C.I. of the drug combination was 0.647, suggesting that this combination has a synergistic effect on U87 MG cells.

### Determination of apoptosis via annexin V-FITC staining

**Fig. 8:**
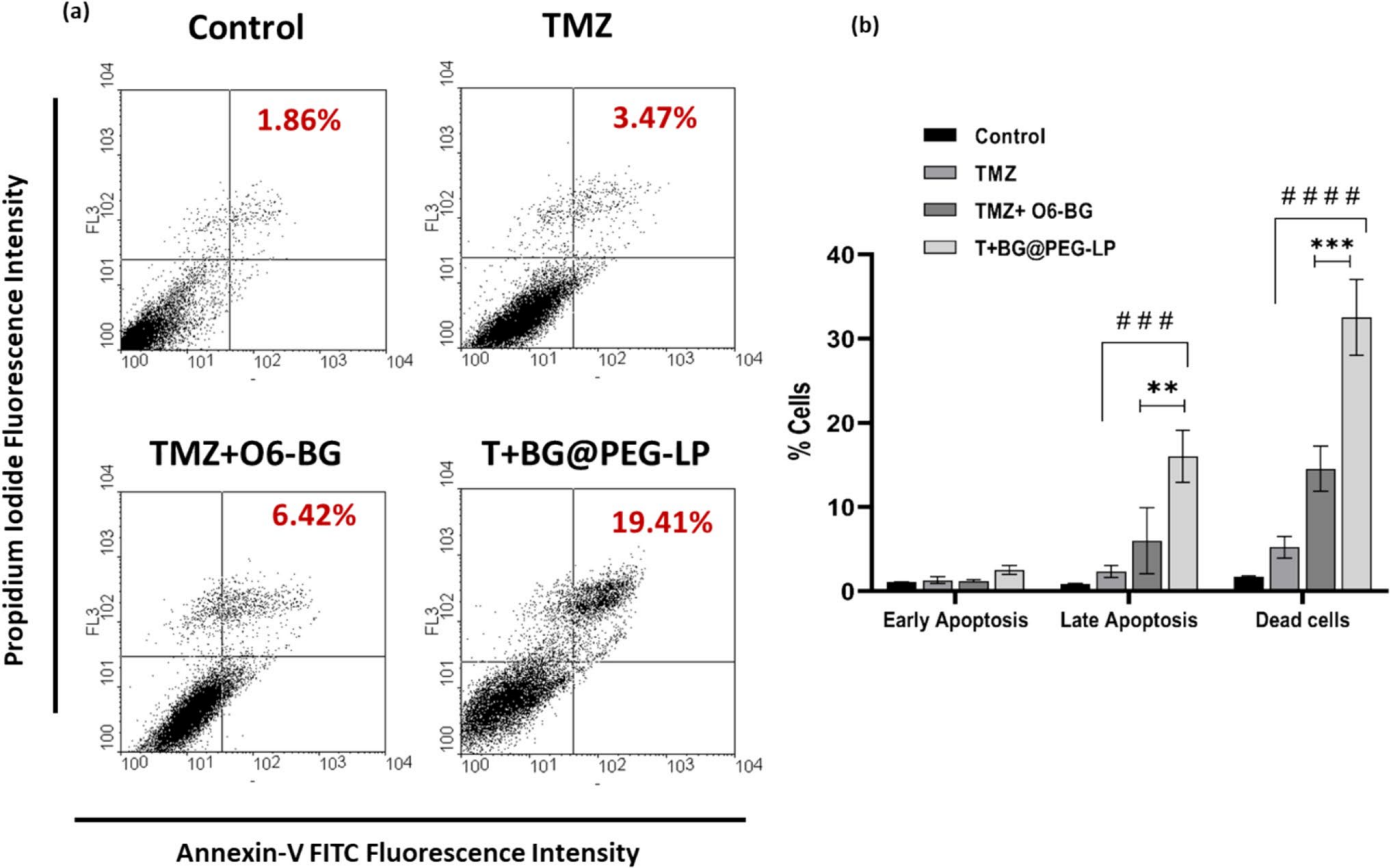
**a** Representative FACS dot plot for U87 MG cells stained with Annexin V-FITC/PI after treatment with pure drugs and drug-loaded liposomes for 72 h. U-87 MG cells showing the significant increase in late apoptotic stage due to sustained release of drugs leading to cell death in those treated with T + BG@PEG-LP in comparison to that of pure TMZ + O6-BG**; b** graph indicating the percentage of cells distributed in each stage of apoptosis. The data are presented as the mean ± SD; the symbols indicate **p < 0.01 and ***p < 0.001 compared with the O6-BG group and ^##^*p* < 0.001 and ^###^*p* < 0.0001 compared with the TMZ group

To determine the induction of cell death due to pure TMZ, TMZ + O6-BG, or different liposomes, apoptosis analysis was performed. The results revealed a toxic effect of T + BG@PEG-LP on U87 MG cells. At 72 h post-treatment, most of the cells were either in the late apoptotic stage or died (Fig. 8). The cells exposed to T + BG@PEG-LP (containing 5 µg/ml TMZ) exhibited sustained death of glioma cells, indicating the controlled release of TMZ from the nanocomposite compared with the pure drug combination (***p < 0.001).

### Influence of liposomal nanocomposites on the cell cycle

**Fig. 9:**
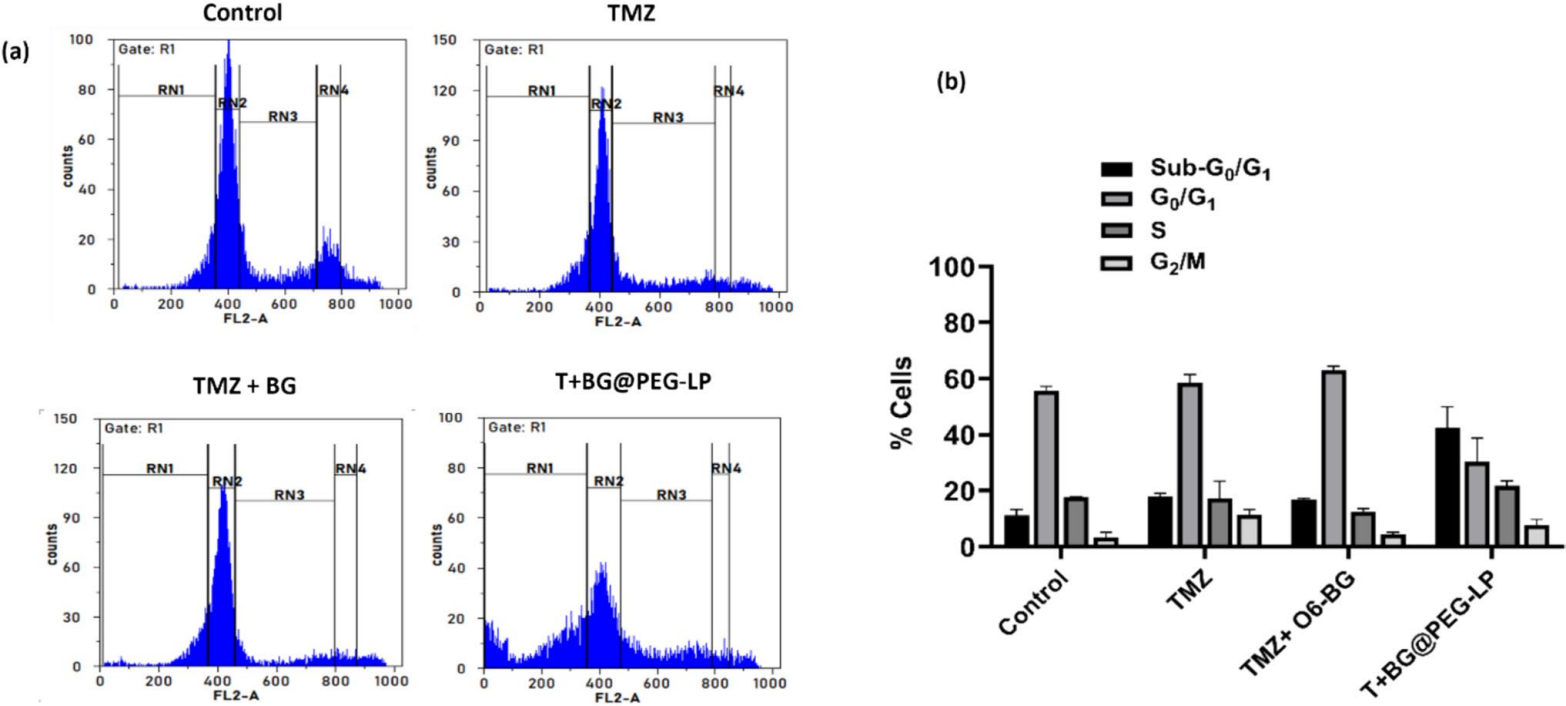
Cell cycle analysis of untreated control, pure TMZ, TMZ + O6-BG, and T + BG@PEG-LP U87 MG cells treated for 72 h. **a** Representative histograms of flow cytometry analysis for cell cycle status; **b** percentage cell distribution at different cell cycle phases showing a significant increase in accumulation of cells at sub-G0/G1 phase, indicating that dual drug-loaded liposomes causing apoptosis by arresting the cells at sub-G0/G1. The data are presented as the mean ± SD

The cell cycle distribution of U87 MG glioblastoma cells was evaluated after treatment with both free TMZ and a combination of TMZ with O6-BG encapsulated in PEGylated liposomes (TMZ + BG@PEG-LP) to assess whether the observed antiproliferative effect was due to apoptosis. There was no significant increase in the sub-G_0_/G_1_ phase after treatment with a combination of free TMZ + O6-BG as opposed to free TMZ alone (Table 3). Our study found a significant increase in cells accumulating in the sub-G0/G1 phase after treatment with T+ BG@PEG-LP compared to the free TMZ treatment, as shown in Fig. 9a and b (42.21 ± 7.02%, *p* < 0.0001). This result suggests a shift in the mechanism of action toward enhanced apoptosis with the liposomal formulation. While TMZ is well-established for causing G2/M arrest in the cell cycle (Ananta et al. 2015; Tentori and Graziani 2009), the combination of TMZ with BG encapsulated in PEGylated liposomes shows a more profound apoptotic effect, as evidenced by the increased accumulation of cells in the sub-G0/G1 phase. This difference suggests that when TMZ is combined with BG in liposomal formulation not only disrupts the cell cycle but also enhances apoptotic pathways more effectively than TMZ alone. The addition of O6-BG, a known inhibitor of the DNA repair enzyme MGMT, likely contributes to the higher apoptotic effect by preventing glioma cells from repairing the DNA damage caused by TMZ. Our findings align with previous studies demonstrating the apoptotic effects of liposomal drug formulations. For example, a recent study by Badivi et al. (2024) reported that PEGylated liposomes used to treat A549 lung cancer cells resulted in a substantial accumulation of cells in the sub-G0 phase after treatment (Badivi et al. 2024). A study by Carmo et al. (2011) showed that TMZ alone could also induce sub-G0/G1 arrest in glioblastoma cells, indicating that TMZ’s ability to promote apoptosis is enhanced under certain conditions, particularly when combined with agents that inhibit DNA repair (CARMO et al. 2011). By facilitating both cell cycle arrest and apoptosis, the T+ BG@PEG-LP formulation demonstrates superior cytotoxicity, which could improve outcomes in glioblastoma treatment. However, a deeper mechanistic approach is needed to understand the molecular pathways involved in inducing sub-G0/G1 arrest due to TMZ + O6-BG combination treatment.

**Table 3:** Percent cell distribution at different cell cycle phases

Treatment groups	% sub-G_0_/G_1_	% G_0_/G_1_	% S	% G_2_/M
Control	11.1 ± 2.04	55.71 ± 1.52	17.01 ± 0.11	3.32 ± 1.72
TMZ	18.07 ± 0.97	58.28 ± 2.95	17.16 ± 6.15	11.47 ± 1.80
TMZ + O6-BG	15.39 ± 1.52	63.47 ± 1.73	12.57 ± 1.52	4.37 ± 0.77
T + BG@PEG-LP	42.21 ± 7.02^+++, ****^	30.295 ± 8.46	21.77 ± 1.66	7.75 ± 2.07

(data are shown as the mean ± SD, n = 3; the symbols indicate ^+++^*p* < 0.001 compared with TMZ and ****p < 0.0001 compared with TMZ + O6-BG)

## Conclusion

In this study, improved TMZ and O6-BG brain delivery via liposomes was proposed. Thus, PEGylated liposomes with suitable physicochemical characteristics for effective brain delivery have been developed. The nanoformulation exhibited a desirable particle size with good drug encapsulation and was able to maintain sustained drug release under physiological conditions. The cytotoxicity results against U87 glioma cells revealed increased antiproliferative activity of TMZ in the presence of O6-BG. Thus, the co-loading of TMZ and O6-BG in PEGylated LPs could be a useful and efficient method for treating glioma because of its focused delivery to tumors, ability to reverse drug resistance, and increased therapeutic efficiency. Even though these NPs may be able to overcome the limits of the treatments that are currently available, future in vivo studies are needed to validate the effectiveness of the nanosystem.

## Supplementary Information

Below is the link to the electronic supplementary material.

Supplementary file1 (DOCX 244 KB)

## Data Availability

All data generated or analyzed during this study are included in this article.
