# Supplementary material for: Enhancing glioblastoma cytotoxicity through encapsulating O6-benzylguanine and temozolomide in PEGylated liposomal nanocarrier: an in vitro study
Source: 3 Biotech. 2024 Oct 23;14(11):275. doi: 10.1007/s13205-024-04123-2 (PMC11499494; doi:10.1007/s13205-024-04123-2)
Supplement: Supplementary file 1 — Supplementary file1 (DOCX 244 KB) [file 13205_2024_4123_MOESM1_ESM.docx]

**Supplementary information**

**Enhancing Glioblastoma Cytotoxicity through Encapsulating O6- Benzylguanine and Temozolomide in PEGylated Liposomal Nanocarrier: An *In vitro* Study**

**Pre-formulation/ drug excipient interaction study**

**Differential scanning calorimetry (DSC):**

To determine the purity of drugs, TMZ and O6-BG were separately sealed in an aluminum pan. The thermograms were recorded for both drugs (from 25°C to 300°C) in a differential scanning calorimeter (DSC 60+, Shimadzu, Japan).

**FTIR analysis:**

Infrared spectra of the pure TMZ, O6-BG, and physical mixture of drugs and lipids were recorded on an FTIR spectrometer (Shimadzu, USA) via the KBr disk method from 4,000 to 500 cm^-1^.

**
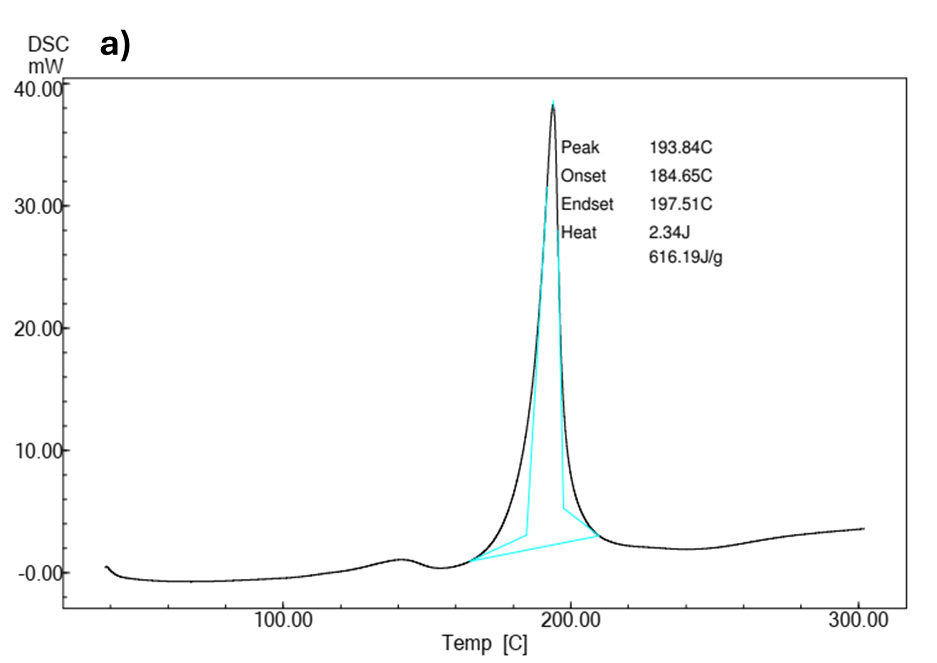
**

**
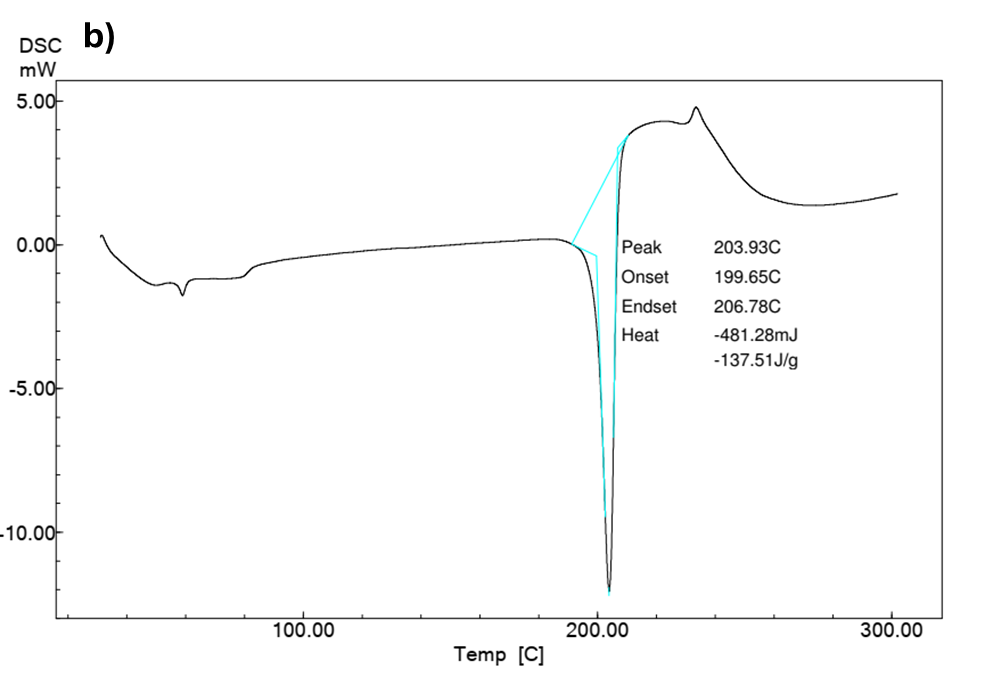
**

**Figure S1:** Representative DSC thermograms of a) TMZ and b) O6-BG.

Thermal analysis of drugs via DSC aids in confirming the purity and crystallinity. A sharp exothermic peak was observed at 193°C for TMZ which is close to the reported peaks (Ji & Liu, 2020; Waghule et al., 2021), and a sharp endothermic peak at 203°C for O6-BG, indicating the purity and crystalline nature of drugs.

**a)**

**b)**

**c)**

**d)**

**Figure S2**: FTIR spectra of a) TMZ, b) O6-BG, c) physical mixture excipients (SPC, DPPC and cholesterol), and d) physical mixture of TMZ, BG, and excipients

The Fourier transform infrared spectrum for pure temozolomide has been recorded with three broad bands at 3387, 1743.65 and 1680 cm^-1^ given by the stretching vibration modes of NH_2_, C=O and OH^-^ groups **(Fig. S2a)** (Wang et al., 2021). In O6-Benzylguanine spectra, 2 sharp peaks were observed at 1633.71 cm^-1^ and 1583.56 cm^-1^ given by C=N and benzene ring, respectively **(Fig. S2b).** Similarly, in the lipid mixtures **(Fig. S2c),** the characteristic bands show the asymmetrical and symmetrical stretching vibration of CH2 of the alkyl chain are seen at 2922 cm-1 and 2858 cm-1, respectively (Dreier et al., 2019). A peak at wave number 1737.86 cm^-1^ corresponds to the aliphatic ester grouping of the soya lecithin. Other bonds at 3336.86 cm^-1^  corresponding to OH stretching of cholesterol (Aisha et al., 2014). The IR spectra of the physical mixture of excipients and drugs retained their characteristic peaks **(Fig. S2d)**. This indicates the physical stability/compatibility and absence of significant interactions between the drugs and excipients used in liposomes.

**References**

Aisha, A. F., Majid, A. M. S. A., & Ismail, Z. (2014). Preparation and characterization of nano liposomes of Orthosiphon stamineusethanolic extract in soybean phospholipids. *BMC Biotechnology*, *14*(1), 23. https://doi.org/10.1186/1472-6750-14-23

Dreier, L. B., Bonn, M., & Backus, E. H. G. (2019). Hydration and Orientation of Carbonyl Groups in Oppositely Charged Lipid Monolayers on Water. *The Journal of Physical Chemistry B*, *123*(5), 1085–1089. https://doi.org/10.1021/acs.jpcb.8b12297

Ji, W., & Liu, Z. (2020). Temozolomide-Loaded Solid Lipid Nanoparticles@Hydrogel for Local Treatment of Tumour. *Australian Journal of Chemistry*, *74*(2), 125–134. https://doi.org/10.1071/CH20018

Waghule, T., Rapalli, V. K., Singhvi, G., Gorantla, S., Khosa, A., Dubey, S. K., & Saha, R. N. (2021). Design of temozolomide-loaded proliposomes and lipid crystal nanoparticles with industrial feasible approaches: Comparative assessment of drug loading, entrapment efficiency, and stability at plasma pH. *Journal of Liposome Research*, *31*(2), 158–168. https://doi.org/10.1080/08982104.2020.1748648

Wang, J., Dai, X.-L., Lu, T.-B., & Chen, J.-M. (2021). Temozolomide–Hesperetin Drug–Drug Cocrystal with Optimized Performance in Stability, Dissolution, and Tabletability. *Crystal Growth & Design*, *21*(2), 838–846. https://doi.org/10.1021/acs.cgd.0c01153
